# Future of information technology and telecommunication in type 1 diabetes clinical care: results of an online survey

**DOI:** 10.1136/bmjdrc-2019-000917

**Published:** 2019-12-31

**Authors:** Daniela Haluza, Samantha Lang, Helen Rogers, Sophie Harris, David Jungwirth, Pratik Choudhary, Ingrid Schuetz-Fuhrmann, Marietta Stadler

**Affiliations:** 1 Department of Environmental Health, Medical University of Vienna, Vienna, Austria; 2 Diabetes Research Group, King’s College London, London, UK; 3 Diabetes, King’s College Hospital, London, UK; 4 3rd Medical Department, Hietzing Hospital Vienna, Vienna, Austria

**Keywords:** type 1, clinical care, patient-physician communication

## Abstract

**Objective:**

To assess the attitude of people living with type 1 diabetes toward the use of information and communication technology (ICT) to facilitate access to diabetes healthcare professionals (HCPs).

**Research design and methods:**

We conducted a cross-sectional online survey in two European tertiary diabetes care centers in London, UK, and Vienna, Austria, and from online diabetes platforms. Participants were asked about general options of online diabetes care and were presented with three scenarios (teleconference, online chat and telemonitoring of continuous glucose monitoring traces).

**Results:**

In total, 294 people (59% female; 78 British, 164 Austrians, 47 Germans, 5 from other countries; 45±15 years) who had been living with type 1 diabetes for 26±14.5 years participated. The vast majority of participants were insulin pump (and/or glucose sensor) users (84%) and reported good glycemic control (31% with hemoglobin A1c (HbA1c) <7% and 51% with HbA1c 7%–8%). ICT was generally acceptable for counseling, with email/online messaging services and online health platform the most preferred options (74% and 53%). Study participants expressed a neutral to positive attitude toward the combined theme scores (relationship with HCP; confidence using technology; trust in data protection; intrusion of patient privacy; general acceptance of ICT in healthcare). UK participants showed more positive attitudes toward ICT across all theme scores than participants from Austria and Germany, but there were no gender-related differences.

**Conclusions:**

This online survey identified a highly ICT-astute group of people with type 1 diabetes, already using technology for insulin delivery, for whom online supported clinical diabetes care would be a viable and welcomed option.

**Significance of this study**

**What is already known about this subject?**

- People living with type 1 diabetes show a preference using communication technology that facilitates communication with healthcare professionals.

**What are the new findings?**

- We found that participants from the UK were more enthusiastic about information and communication technology (ICT) use in diabetes care than those from Austria/Germany.
- Men and women differed in their preferences regarding diabetes data management and online interaction with healthcare professionals.

**How might these results change the focus of research or clinical practice?**

- Targeted, user-centric, ICT-based diabetes care offers valuable features from health service user perspective which could be immediately translated into clinical practice.

## Introduction

Type 1 diabetes mellitus requires lifelong intensive day-to-day self-management involving glucose monitoring, insulin injections adjusted to carbohydrate intake, exercise and illness, carbohydrate counting, and self-motivational skills.^1^ People with type 1 diabetes need regular face-to-face clinical encounters for advice, often necessitating time away from work and significant travel time.^2^ Health technology (smartphone applications, websites, computerized recordings of blood glucose monitoring and online support forums) may facilitate daily disease management. Satisfaction with these technologies is considerable, with the most influential features being healthcare professional (HCP) involvement, facilitating feedback on diabetes self-management from a trained professional, and a sense of feeling supported in diabetes self-management.^3^ Online clinic appointments and patient professional online platforms could provide a convenient, cost-effective way for people to access specialist advice.

Previous, smaller studies have evaluated the attitudes and acceptability of using online interaction with HCPs, but only as part of testing devices and communication methods rather than as the main focus.^4 5^ Some people with diabetes would value a virtual clinic site, stating that ‘Ask an Expert’ or ‘peer support features’ would be the most useful.^6^ Online interventions such as HCP online monitoring of blood glucose with text message and online appointment advice services have been shown to be effective at reducing patient hemoglobin A1c (HbA1c) levels,^7^ but also raise concerns regarding privacy and online data protection.^8^ No study has previously assessed all of these domains in a group of people with type 1 diabetes, whose clinical care takes place within a hospital clinic.

In this study we report on attitudes of people with type 1 diabetes toward online communication with HCPs across two European tertiary diabetes care centers in London, UK, and Vienna, Austria. We assessed attitudes toward online care across five themes, namely relationships with HCPs, privacy, practical benefits, worries around data protection and confidence in technology use, also introducing three potential scenarios providing a realistic impression of real-life information and communication technology (ICT) use.

## Participants and methods

We conducted a cross-sectional online survey among a sample of people receiving diabetes care in two large tertiary diabetes centers: King’s College Hospital (KCH) in London, UK; and Hietzing Hospital Vienna (HHV), in Vienna, Austria. All people with type 1 diabetes attending an outpatient appointment within 2 years of the start of the study were invited to participate. Inclusion criteria were being at least 18 years of age and having a diagnosis of type 1 diabetes for more than 6 months. Patients were excluded if they had gestational diabetes, type 2 diabetes or were participating in other ongoing diabetes studies.

An online link sent to all participants (HHV: direct; KCH: after initial postal contact) enabled access to the online participant information and the study questionnaire between 24 July 2015 and 1 March 2016 via the software package SoSci Survey.^9^ The link to the survey was also posted on social media platforms and on the mySugr user platform.^10^

Participants were informed about the purpose of the study. Survey participation was voluntary and anonymous; no identifying information was collected. Consent was provided by checking a tick box at the start of the study. Source of referral to the study was collected to allow subgroup analysis. The collected data were stored securely and protected from unauthorized access.

The online survey link was clicked 637 times. The questionnaire was partly completed 380 times and fully completed 294 times, resulting in a response rate of 60% and a full cooperation rate of 47%. Participants took a mean±SD of 16.7±5.5 min (range 4.7–31.8 min) to fill in the survey online. KCH sent out 474 invite letters and HHV (IS-F) 308 invite emails. Retrospectively, we excluded three participants who were younger than 18 years, resulting in a final data set of 294 completed questionnaires.

We developed a study questionnaire in English, which was then translated into German by a native speaker (MS) using forward and backward translation. At KCH, one of the authors (SL) attended a voluntary focus group of people with type 1 diabetes to ensure the study’s acceptability and feasibility among its target group.

The online questionnaire consisted of 47 items. Seven sections assessed (1) sociodemographic characteristics and general information, (2) diabetes history, (3) diabetes service use, (4) current information technology (IT) usage, (5) HCP interaction, (6) anonymity and data protection and (7) scenarios. In the questionnaire, items in sections 5–7 were assigned to one of the following themes: relationship with HCP will improve (relationship score), confidence using technology (technology score), trust in data protection (data protection score), worry about intrusion of the patient’s privacy (privacy score), and general acceptance of ICT (acceptance score).

These themes were not disclosed to study participants to avoid bias. The items assessing these themes were validated by three researchers blinded to the coding of the questions (ie, which theme they belonged to); those questions with discordant coding were redesigned and revalidated. A pretest of 20 volunteers assessed the usability and comprehensibility of the survey, and we adapted items according to this feedback.

The three scenarios, video consultation, online chat, and 24/7 remote continuous glucose monitoring system (CGMS), represented possible ways of using ICT in a patient–HCP interaction. All scenarios were already technologically feasible and had been used in clinical studies or as pilot trials for real-life clinical services (online supplementary material table S1).^11–13^

10.1136/bmjdrc-2019-000917.supp1 Supplementary data

We analyzed fully completed questionnaires with less than 5% missing values. For the majority of items, we used 5-point Likert scales ranging from 1=strongly disagree to 5=strongly agree. Descriptive data were presented as mean, median, SD, and percentage. Values not adding up to 100% are due to missing data. Relationship, technology, trust, privacy and acceptance scores were obtained by summing up the answers to the items targeted at that theme. The combined score included summed responses to all items of the themes. Cronbach’s alpha assessed the reliability and internal consistency of the scales.

χ² tests and t-tests assessed subgroup differences for country, digital age groups, and gender regarding categorical and ordinal variables. For country-specific differences, we compared participants from the UK with those from the German-speaking countries, that is, Austria (AUT) and Germany (GER), reflecting the study questionnaire language options German and English and assuming similar attitudes among patients with diabetes in the respective countries. The UK group (n=78, 56% female) consisted of 85% (n=66) participants recruited by KCH and 14% (n=12) participating via online platforms, whereas the AUT/GER group (n=211, 61% female) consisted of 75% (n=158) participants recruited by HHV and 25% (n=53) participating via online platforms. For digital age-specific differences, we followed the definitions coined by Prensky^14^ with digital natives being those ‘born digital’ and born in or after 1980, whereas digital immigrants are defined as those born before 1980, as defined by Palfrey and Gasser.^15^ We conducted a subgroup analysis by educational level to compare the theme scores between these groups, using analysis of variance and post-hoc Bonferroni.

Data were processed and analyzed using Excel (Microsoft, Seattle, Washington) and SPSS V.23. The two-tailed level of statistical significance was set at 0.05.

## Results

In total, 294 patients completed our survey. Of these, 211 (72%) participants were from Austria (164) or Germany (47), 78 (27%) from the UK, and 5 (2%) had other nationalities: Switzerland (n=2), Barbados, Spain, and USA (1 each). All of the non-UK, non-Austrian participants indicated they had heard about the survey via online platforms. Participants (N=294, 60% female) were 44.9±15.0 years old (range 19–85 years). The majority were digital immigrants (n=206, 70%); 88 individuals (30%) were digital natives. The majority of respondents had a university degree (36.%) or higher education certificate (28%), 22% had A-levels or equivalent and 10% had completed secondary school, and only 1% with primary school education.

Overall, 84% indicated daily ICT use for on average just below 5 hours daily, spread evenly across mobile devices (mean±SD 1.7±0.4 hours) and home internet (mean±SD 1.7±0.4 hours). Work internet use was marginally less at a mean±SD of 1.4±0.5 hours.

Participants had been living with diabetes for 26.0±14.5 years (range 2–69 years). The vast majority were insulin pump users (84%), 22% of whom also used CGMS, followed by patients applying multiple daily insulin injection therapy. More than half (51%) stated that their latest HbA1c was between 7.0% and 8.0%, 31% below 7.0%, and only 14% reported an HbA1c above 8%. The vast majority (81%) stated that they had no diabetes-related complications, 14% declared one, 4% two, and 1.4% three diabetes complications. Nine per cent had experienced a diabetes-related inpatient hospital stay (1% more than five stays, 1% three, 2% two and 5% one stay) in the anteceding 12 months. The majority of participants agreed (strongly) that their diabetes is well controlled (71%) and their health is good (69%). Most participants reported to contact their HCP less than once per month (64%), whereas 25% did so two to four times per month and 11% weekly or more often. Most (84%) used ICT daily in their personal and work life, and a third for more than 3 hours per day.

A majority (69%) stated a preference for at least half of their diabetes care to be delivered by online counseling. Online messaging services for general queries concerns and a diabetes profile on an online health platform were the most preferred options (74% and 53%), while video conferencing (31%) was the least preferred (table 1).

**Table 1:** Online counseling preferences and subgroup differences (country, digital age, gender). Data are presented as number of participants (%)

	Country			Digital age			Gender			Total N=294
	UK n=78	Austria/Germany n=211	P value	Digital natives n=88	Digital immigrants n=206	P value	Female n=174	Male n=118	P value	
Healthcare professional contact preferred frequency										
≥1/week	10 (12.8)	21 (10.0)	0.8	11 (12.5)	20 (9.7)	0.1	27 (15.5)	4 (3.4)	0.001	31 (10.7)
2–4/month	19 (24.4)	53 (25.1)		28 (31.8)	45 (21.8)		50 (28.7)	22 (18.6)		73 (25.1)
<1/month	48 (61.5)	135 (64.0)		49 (55.7)	138 ((67.0)		97 (55.7)	89 (75.4)		187 (64.3)
Individual vs group interaction online										
One-to-one	49 (62.8)	129 (61.1)	1.0	50 (56.8)	132 (64.1)	0.02	103 (59.2)	78 (66.1)	0.5	182 (64.1)
Group	1 (1.3)	2 (0.9)		3 (3.4)	0 (0.0)		2 (1.1)	1 (0.8)		3 (1.1)
Either/both	26 (33.3)	72 (34.1)		34 (38.6)	65 (31.6)		63 (36.2)	35 (29.7)		99 (34.9)
Video conferencing										
Yes	28 (35.9)	59 (28.0)	0.2	26 (29.5)	64 (31.1)	0.8	48 (27.6)	42 (35.6)	0.2	90 (30.6)
No	50 (64.1)	152 (72.0)		62 (70.5)	142 (68.9)		126 (72.4)	76 (64.4)		204 (69.4)
Online messaging										
Yes	70 (89.7)	134 (67.8)	<0.001	70 (79.5)	148 (71.8)	0.2	138 (79.3)	79 (66.9)	0.02	218 (74.1)
No	8 (10.3)	68 (32.2)		18 (20.5)	58 (28.2)		36 (20.7)	39 (33.1)		76 (25.9)
Text messaging										105 (35.7)
Yes	38 (48.7)	65 (30.8)	0.01	33 (37.5)	72 (35.0)	0.7	73 (42.0)	32 (27.1)	0.01	105 (35.7)
No	40 (51.3)	146 (69.2)		55 (62.5)	134 (65.0)		101 (58.0)	86 (72.9)		189 (64.3)
Online feedback of self-monitoring										
Yes	47 (60.3)	94 (44.5)	0.01	49 (55.7)	96 (46.6)	0.2	96 (55.2)	49 (41.5)	0.02	145 (49.3)
No	31 (39.7)	117 (55.5)		39 (44.3)	110 (53.4)		78 (44.8)	69 (58.5)		149 (50.7)
Continuous glucose remote monitoring										
Yes	34 (43.6)	57 (27.0)	0.01	27 (30.7)	65 (31.6)	0.9	53 (30.5)	39 (33.1)	0.6	92 (31.3)
No	44 (56.4)	154 (73.0)		61 (69.3)	141 (68.4)		121 (69.5)	79 (66.9)		202 (68.7)
Online diabetes profile										
Yes	45 (57.7)	107 (50.7)	0.3	50 (56.8)	106 (51.5)	0.4	94 (54.0)	60 (50.8)	0.6	156 (53.1)
No	33 (42.3)	104 (49.3)		38 (43.2)	100 (48.5)		80 (46.0)	58 (49.2)		138 (46.9)
No healthcare professional contact online										
Yes	2 (2.6)	19 (9.0)	0.07	5 (5.7)	16 (7.8)	0.5	11 (6.3)	10 (8.5)	0.5	21 (7.1)
No	76 (97.4)	192 (91.0)		83 (94.3)	190 (92.2)		163 (93.7)	108 (91.5)		273 (92.9)

Values not adding up to 100% are due to missing data.

Table 2 depicts the scores for the five themes of online interaction. The levels of all scores were moderate, with the median reaching 50%–64% of the respective maximum score: relationship score 55%, technology score 50%, data protection score 55%, privacy score 60%, general acceptance score 64%, and combined score 59%. Measures of reliability varied between the scores of the themes: relationship score alpha=0.597, technology score alpha=0.745, data protection score alpha=0.694 (all: 4 items), privacy score alpha=0.554 (3 items), and general acceptance score alpha=0.604 (11 items), with good internal consistency for the combined score (alpha=0.889, 26 items). All scenarios showed good internal reliability: video conference alpha=0.824 (6 items), remote CGMS alpha=0.824 (7 items), and online chat alpha=0.890 (6 items). Participants were, on average, neutral in their confidence using ICT, found IT generally acceptable, and were neutral with regard to worries regarding intrusion on privacy and trust in data protection.

**Table 2:** Scores of online interaction themes and subgroup differences (country, digital age, gender)

	Relationship score	Technology score	Data protection scrore	Privacy score	General acceptance score	Combined score
Number of items	4	4	4	3	11	26
Minimum score	4	4	4	3	11	26
Maximum score	20	20	20	15	55	130
Mean	11.78	11.63	12.07	8.71	35.36	79.80
Median	11	10	11	9	35	76
SD	3.65	4.35	3.84	3.04	6.41	18.01
Range, minimum	4	4	4	3	11	32
Range, maximum	20	20	20	15	54	127
Country						
UK						
Mean	15.16	16.81	15.55	10.29	39.42	97.23
SD	2.57	2.71	3.25	2.08	7.23	15.48
Austria/Germany						
Mean	10.47	9.65	10.73	8.13	33.78	72.97
SD	3.08	2.98	3.13	3.13	5.23	13.57
P value	0.001	0.001	0.001	0.001	0.001	0.001
Digital age						
Digital natives						
Mean	11.47	10.86	11.09	8.47	34.42	76.64
SD	3.16	4.37	3.90	3.13	6.46	17.82
Digital immigrants						
Mean	11.92	11.96	12.49	8.82	35.75	81.15
SD	3.83	4.31	3.74	3.01	6.36	17.97
P value	0.3	<0.05	0.004	0.4	0.1	0.05
Gender						
Female						
Mean	11.85	11.44	11.87	8.85	35.24	79.44
SD	3.41	4.20	3.87	3.10	6.03	17.45
Male						
Mean	11.60	11.85	12.36	8.49	35.39	80.03
SD	3.93	4.56	3.82	2.99	6.87	18.85
P value	0.6	0.4	0.3	0.3	0.8	0.9
Education						
Primary education (a)						
Mean	12.3	12.1	12.2	9.2	36.1	81.8
SD	3.9	4.4	3.9	2.7	5.8	17.9
Secondary education (b)						
Mean	11.1	10.7	11.4	8.2	34.4	76.2
SD	3.4	3.8	3.5	3.2	5.9	16.4
Tertiary education (c)						
Mean	12.5	12.7	13.0	9.2	36.4	84.0
SD	3.7	4.8	4.0	2.9	7.2	19.4
P value, ANOVA	0.08	0.02	0.05	0.02	0.04	0.03
P value, post-hoc	0.08, c vs b	<0.001, c vs b	0.001, c vs b	0.03, c vs b	<0.05, c vs b	0.002, c vs b

ANOVA, analysis of variance.

The scenarios were perceived as neutral to slightly positive (video conference (3.1; 2.8–3.3), remote CGMS (3.1; 2.4–3.3), and online chat (3.2; 2.9–3.4), score range 1–5), although there were concerns regarding data upload and CGMS use. Video clinics were preferred to face-to-face clinics by 39%, whereas 33% would be more likely to contact their HCP with problems if an online chat facility existed, and 34% agreed that they would use remote CGMS if they were to develop problematic hypoglycemia.

### Subgroup analyses

Participants from the UK were more likely to have internet at home and at work, but in both countries more than 70% had internet access via a mobile device (all: p<0.05; table 3). Participants from AUT/GER were more likely to use an insulin pump and were downloading glucometer, pump or CGMS data more often than their English counterparts (all: p<0.05; table 4). Despite this, participants from AUT/GER were more reticent to engage with HCPs online, with differences regarding online messaging, text messaging, online feedback of self-monitoring, and CGMS remote monitoring (all: p<0.05; table 1). Participants from AUT/GER were younger (42.9±14.6 years) than the UK participants (50.8±14.4 years, p<0.001), resulting in a higher proportion of digital natives (33% vs 15%, p=0.001).

**Table 3:** IT usage in different day-to-day settings plus IT use frequency by country, digital age, and gender, n (%)

	Country			Digital age			Gender			Total N=294
	UK n=78	Austria/Germany n=211	P value	Digital natives n=88	Digital immigrants n=206	P value	Female n=174	Male n=118	P value	
Daily IT usage										
Yes	66 (84.6)	177 (83.9)	0.880	85 (96.6)	163 (79.1)	0.001**	146 (83.9)	101 (85.6)	0.696	248 (84.4)
No	12 (15.4)	34 (16.1)		3 (3.4)	43 (20.9)		28 (16.1)	17 (14.4)		46 (15.6)
Internet at home										
Yes	76 (97.4)	137 (64.9)	0.001**	54 (61.4)	163 (79.1)	0.002*	116 (66.7)	99 (83.9)	0.001**	217 (73.8)
No	2 (2.6)	74 (35.1)		34 (38.6)	43 (20.9)		58 (33.3)	19 (16.1)		77 (26.2)
Internet at work										
Yes	39 (50.0)	67 (31.8)	0.004*	34 (38.6)	76 (36.9)	0.777	56 (32.2)	54 (45.8)	0.019*	110 (37.4)
No	39 (50.0)	144 (68.2)		54 (61.4)	130 (63.1)		118 (67.8)	64 (54.2)		184 (62.6)
Mobile internet access										
Yes	56 (71.8)	159 (75.4)	0.538	84 (95.5)	136 (66.0)	0.001**	136 (78.2)	84 (71.2)	0.175	220 (74.8)
No	22 (28.2)	52 (24.6)		4 (4.5)	70 (34.0)		83 (21.8)	34 (28.8)		74 (25.2)

Values not adding up to 100% are due to missing data.

*P<0.05, **P<0.01.

IT, information technology.

**Table 4:** Current usage of diabetes-related technology subgroup analysis and acceptability of different modes of interaction by country, digital age, and gender, n (%)

	Country			Digital age			Gender			Total N=294
	UK n=78	Austria/Germany n=211	P value	Digital natives n=88	Digital immigrants n=206	P value	Female n=174	Male n=118	P value	
DM therapy										
Multiple daily insulin injection	23 (29.5)	25 (11.8)	0.001	19 (21.6)	31 (15.0)	0.2	37 (21.4)	13 (11.2)	0.02	48 (16.4)
Insulin pump therapy	55 (70.5)	186 (88.2)		69 (78.4)	175 (85.0)		137 (79.2)	105 (90.5)		244 (83.0)
Glucometer download										
<3/month	55 (70.5)	111 (52.6)	0.01	62 (70.5)	105 (51.0)	0.03	111 (64.2)	55 (47.4)	0.02	167 (60.7)
1–3/month	8 (10.3)	49 (32.2)		14 (15.9)	45 (21.8)		33 (19.1)	25 (21.6)		59 (21.5)
>1/ month	8 (10.3)	40 (19.0)		10 (11.4)	39 (18.9)		22 (12.7)	27 (23.3)		49 (17.89
Pump/CGMS download										
<3/month	47 (60.3)	105 (49.8)	0.3	58 (65.9)	96 (46.6)	0.01	104 (60.1)	50 (43.1)	0.01	154 (52.6)
1–3/month	19 (24.4)	60 (28.4)		20 (22.7)	60 (29.1)		39 (22.5)	40 (34.5)		80 (27.3)
>1/ month	12 (15.4)	45 (21.3)		10 (11.4)	49 (23.8)		31 (17.9)	28 (24.1)		59 (20.1)
Email download										
<3/month	73 (93.6)	181 (85.8)	0.1	74 (84.1)	185 (89.8)	0.2	155 (89.6)	102 (87.9)	0.9	259 (89.0)
1–3/month	3 (3.8)	24 (11.4)		12 (13.6)	15 (7.3)		15 (8.7)	12 (10.3)		27 (9.3)
>1/ month	1 (1.3)	4 (1.9)		2 (2.3)	3 (1.5)		3 (1.7)	2 (1.7)		5 (1.7)

CGMS, continuous glucose monitoring system; DM, diabetes mellitus.

This reluctance was also seen when analyzing the five themes of online interaction, assessing relationship with HCPs online, trust in data protection, confidence with technology, concerns regarding personal privacy and general acceptance of health technologies online (table 2). UK participants showed higher approval for all themes (all: p<0.001) compared with participants from AUT/GER. UK participants also showed more positive attitudes toward HCP interaction online, a higher confidence in technology use, and were generally more accepting of integrating technology into their diabetes healthcare management. AUT/GER participants were more concerned about their online data protection and invasion of their privacy when using online health technologies than UK participants.

Digital natives were more likely to use the internet on a daily basis, access the internet on a mobile device compared with digital immigrants (both: p<0.001) and have internet at home (p<0.05; table 3). Digital natives were less likely to use technologies related to their diabetes care (insulin pump therapy, glucometer and CGMS downloads, all: p<0.05; table 4). The majority of participants downloaded their glucometer and pump less often than every 3 months (61% and 53%); this proportion was higher in the digital native group (71%). With regard to interacting with HCPs online, digital natives were less concerned regarding interacting with a group rather than on an individual 1:1 capacity compared with digital immigrants (p<0.05).

Digital natives consistently scored lower across all theme scores, reaching significance for the technology score (mean 10.9, SD 4.4 vs mean 12.0, SD 4.3) as well as the data protection score (mean 11.1, SD 4.0 vs 12.5, SD 3.7, both: p<0.05; table 2). Compared with digital immigrants, digital natives were, despite a higher IT use (table 3), less confident at using IT and also more worried regarding data protection issues (table 2). However, the two groups were similar in their attitudes toward online HCP interaction (table 1).

Both genders had a similar proportion of daily IT usage, but more men than women had internet access at home and work (both: p<0.05; table 3). Men were more likely to use a pump and to download diabetes data from glucometer, pump and CGMS more frequently (all: p<0.05; table 4). Female participants had a higher preference for more frequent online interaction with HCPs via online messaging, instant text messaging and online messaging and feedback on blood glucose reading downloads (all: p<0.05; table 1). Female and male participants yielded similar scores for all themes (table 2).

Participants with tertiary level education scored higher on all theme scores than people with secondary education (table 2).

## Discussion

This online survey among adults living with type 1 diabetes analyzed prevailing attitudes and modes of telecare in the context of the patient–web–physician triangle.^16^ The respondents, who were recruited from large tertiary type 1 diabetes outpatient services, were astute ICT users in their daily life, with the majority using advanced diabetes technology to manage their diabetes. The vast majority had good glycemic control and no diabetes complications in spite of living with diabetes for on average more than 25 years, suggesting a high level of commitment to diabetes self-care.

In general, attitudes toward ICT-mediated diabetes care delivered by their diabetes care teams were positive, which was reflected in a readiness to engage with all proposed modalities of ICT, including the three plausible clinical scenarios described. In comparison with studies in the general population, people living with diabetes are likely to have lower thresholds of engaging with technology for their self-care.

Technology acceptance differs between expert groups representing medical professionals, patient advocates and administrative personnel.^17–19^ Thus, closer cooperation between related stakeholders is vital for successful IT implementation and adoption. Also, readiness for telemonitoring was shown to be higher among patients compared with HCPs.^20^

Despite higher diabetes technology use, participants from the German-speaking countries were less enthusiastic about using ICT in their diabetes care than those in the UK. There is a potential cultural background to this finding, as Austrian internet users were shown to be skeptical regarding health data electronic exchange between HCPs and patients.^21 22^ It is also likely that differences in access to tertiary healthcare between UK and AUT/GER^23^ influence the readiness to engage in ICT online diabetes care.

This study has several limitations. The response rate was quite low and there were more participants from Austria than London. While attempts were made to maximize the response rate by sending out multiple reminders to participate, the findings are not generalizable to all people with type 1 diabetes. Also, the cross-sectional study design did not allow determining temporal order, directions and causalities of observed associations. Only people with internet access could participate in this study. However, we strove at collecting information from current users of digital health technologies to assess their opinions and future care requirements.

This study identified a motivated group of people with type 1 diabetes, already using technology for insulin delivery, for whom, given their low threshold for ICT use and well-managed diabetes, online clinical care could be a viable and welcomed option. Closing the knowledge gap on health IT-related patients’ expectations and opinions, this study adds so far lacking information on country-specific, gender-specific, and digital age-specific divergences regarding the future direction of diabetes type 1 care in an increasingly digitized health system. This may contribute to guidance and policy-making regarding the future applications of clinical technology in caring for people with diabetes.
