# Supplementary material for: Future of information technology and telecommunication in type 1 diabetes clinical care: results of an online survey
Source: BMJ Open Diabetes Res Care. 2019 Dec 31;7(1):e000917. doi: 10.1136/bmjdrc-2019-000917 (PMC6954756; doi:10.1136/bmjdrc-2019-000917)
Supplement: Supplementary data [file bmjdrc-2019-000917supp001.pdf]

Suppl. Table 1 Scenarios

Scenario 1. Video-conference clinic

Your diabetes clinic offers you an online video appointment (e.g. Skype/Facetime) with a health care professional for a specific time and date. This video appointment allows you to see and speak to your diabetes doctor/nurse about your diabetes and your blood tests like a normal clinic appointment.

For your video appointment you can be at home, at work, wherever is convenient for you (as long as you have access to a webcam, computer and the internet).

You are asked to download your glucometer readings at home from your glucometer to your computer, or scan your diabetes diaries and put/upload these readings onto a secure hospital internet program so that your diabetes doctor/nurse can see them during your video appointment. Your GP is asked to upload the results of your recent blood tests as well.

Please, let us know how you would feel about attending a video clinic.

How strongly would you agree with the following statements? (Strongly agree, agree, neutral, disagree, strongly disagree)

I am fine with communicating with my doctor or nurse over a video-screen instead of seeing them in person.

I do not think that this would change the relationship between me and the health care professional.

A video clinic would be easier for me to fit into my busy life.

I would feel confident uploading data if someone shows me how to do that.

I am confident that a hospital online programme would protect my health data appropriately.

I general, I would prefer video clinics over face-to-face clinic visits.

I would not mind my doctor/nurse seeing my work or home environment on the video screen.

Scenario 2. Continuous Glucose Monitoring

Consider this scenario: Your sugar levels are like a roller coaster at the moment and you have had several episodes of very low blood sugar (severe hypoglycaemia) in the last year that had to be treated with the help of others (this is just an assumption for this scenario). Your diabetes team is offering to "keep an eye" on your glucose levels by giving you a glucose monitoring system you wear on your body all the time and that directly sends the glucose readings to a monitor in the diabetes clinic.

The screen is being watched by a health care professional (diabetes doctor/nurse) at all times and an alarm is set off if your glucose levels go high, very low, or if there are no data transmitted. If an alarm is set off, the diabetes team gets in contact with you to see if you need any help, or sends you an ambulance if you don't respond and the glucose seems very low on their monitor.

Please, let us know how you would feel about continuous remote monitoring of your glucose, if you were a patient who is suffering from severe hypoglycaemia.

How strongly would you agree with the following statements?
(Strongly agree, agree, neutral, disagree, strongly disagree)

I anticipate an adequately trained person to look at the glucose monitoring screen and I would trust that person to give me good advice.

I feel that this monitoring could help to prevent severe hypoglycaemic episodes and could avoid unnecessary hospital stays.

I would feel confident using a glucose monitoring device if someone shows me how to do that.

I am confident that my glucose levels will only be observed by the *health care professional* in charge of supporting me.

I would use such a service, if I were someone with problematic hypoglycaemia.

I would be concerned about someone knowing my glucose levels at all times (e.g. when I eat, exercise or travel, etc.).

I would be willing to pay something for this additional service.

Scenario 3. Online glucose chat:

Consider this scenario: You have been feeling unwell today with a bad cold and now your blood glucose is running high. You are in a bus on the way home from work and don't feel like phoning the hospital switchboard to speak to your diabetes doctor/nurse, but you have your smartphone with you. Your diabetes clinic has given you the link to an online chat service where you can send and receive typed messages between a diabetes doctor/nurse. You can use this for urgent questions, when you do not feel well or you feel unable to manage your blood sugars alone. There is a 24 hours a day, seven days per week access to a member of the diabetes team via this service.

Please, let us know how you would feel about using an online glucose chat platform to communicate with your diabetes team for urgent questions.

How strongly would you agree with the following statements?
(Strongly agree, agree, neutral, disagree, strongly disagree)

I anticipate an adequately trained person to answer the chat and trust that person is qualified to help me

The online chat service would make me more likely to contact my diabetes doctor/nurse when I am in trouble with my diabetes

I would feel confident using an online chat if someone shows me how to do that

I am confident that the content of the chat will remain confidential

I would not mind if the person sitting next to me in the bus would see that I am chatting about my diabetes on my smartphone

I would use the glucose online chat for urgent questions on my diabetes treatment rather than the telephone
